# Time-varying reliability analysis of the main arch ring in reinforced concrete arch bridges considering non-stationary degradation

**DOI:** 10.1371/journal.pone.0336613

**Published:** 2025-11-17

**Authors:** Yue Guo, Zhuo Li, Ke Xie, Yuanpeng Luo, Ziwei Wang, Wan Zhao, Zhongchu Tian

**Affiliations:** 1 Bridge Engineering Branch Company, Sichuan Road & Bridge(Group) Co., Ltd., Chengdu, Sichuan, China,; 2 Engineering Management Department, Sichuan Chuanqian Expressway Co., Ltd., Chengdu, Sichuan, China; 3 School of Civil and Environmental Engineering, Changsha University of Science & Technology, Changsha, Hunan, China; Guangdong University of Petrochemical Technology, CHINA

## Abstract

Reinforced concrete arch bridges are susceptible to non-stationary degradation under combined environmental and load effects, rendering traditional reliability assessments based on stationary assumptions inadequate. To address this gap, this study first derived a reliability calculation method tailored for non-stationary degradation scenarios. Subsequently, an ISSA-Kriging surrogate model was proposed for the reliability evaluation of reinforced concrete arch bridges, with validation and analysis conducted using the Shuiluo River Bridge as an engineering case. Results indicate that the ISSA-Kriging model achieves high prediction accuracy: its sample response error is controlled within 4% in repeated random sampling tests, and its accuracy is approximately 60% higher than that of the standard Kriging model. The model reliably fits the time-varying reliability curve of the main arch ring, confirming its suitability for large-scale parametric analysis and engineering optimization. Compared with stationary degradation, non-stationary degradation accelerates the decay rate of the main arch ring’s reliability index by 20%–30%. After 50 years of service, the reliability reduction rates of the arch springing, arch crown, and mid-span (1/2 arch ring) under non-stationary degradation reach 90.8%, 97.8%, and 52.7%, respectively, leading to an obvious “unimodal” reliability distribution across the semi-structure of the main arch ring. Additionally, non-stationary load fluctuations exacerbate structural damage accumulation, emphasizing the need for targeted durability protection of key components. A limitation of this study is that the proposed non-stationary degradation model, while theoretically consistent with non-stationary deterioration laws and validated via numerical simulation, lacks direct calibration with long-term on-site monitoring data. Future research will focus on integrating structural health monitoring data to dynamically revise the model, narrowing the gap between numerical simulation and actual structural performance, and thereby enhancing the engineering practical value of non-stationary reliability assessment results. This study provides a robust technical tool for the non-stationary reliability assessment of reinforced concrete arch bridges and offers guidance for durability design and maintenance optimization.

## 1. Introduction

Reinforced concrete arch bridge is an important part of transportation infrastructure [1], with the advantages of large span capacity, high bearing capacity and good durability, it has been widely used in bridge engineering [2,3]. However, in the process of long-term service, the arch ring structure is affected by multiple factors such as environmental erosion, load and material aging, and its performance presents complex time-varying characteristics [4,5]. The traditional reliability analysis is based on the assumption of stable degradation, that is, the degradation rate of structural performance is constant, a large number of engineering practices show that the deterioration process is often non-stationary – for example, the corrosion rate may be accelerated due to the cracking of the protective layer, or the temperature and humidity changes show seasonal fluctuations [6–9]. This non stationarity poses a major challenge to the time-varying reliability assessment, and is also a key problem to be solved in the field of structural safety assessment [10–12].

To tackle the above problems, early studies mainly focused on static reliability analysis, ignoring the influence of the time dimension. With the gradual development of time-varying reliability theory, it is still difficult to accurately reflect the actual degradation law by assuming that the deterioration process is a stationary stochastic process or using simplified linear degradation models. Some scholars have attempted to introduce non-stationary stochastic processes to describe the deterioration behavior of arch bridges, but research on the nonlinear deterioration mechanism and time-varying reliability of arch bridge rings remains insufficient. In addition, as a composite component subjected to both compression and bending, the arch ring of arch bridges exhibits significant spatial variability in its degradation mode, which further increases the complexity of analysis [13–15].

Based on relevant literature, there are still many research gaps in the study of reinforced concrete arch bridges, and further in-depth research is needed regarding the degradation mechanism and reliability calculation of reinforced concrete arch rings. Therefore, this paper proposes a time-varying reliability analysis method for reinforced concrete arch bridge rings considering non-stationary deterioration. A non-stationary stochastic process is adopted to describe the degradation law of arch ring resistance. Combined with time-varying reliability theory, the limit state equation for the bearing capacity of the arch ring is established. The influence of non-stationary deterioration on the structural failure probability is quantified through the probability density evolution method. Finally, the applicability of the proposed model is verified by integrating theoretical calculation data from engineering cases and finite element models, providing support for the life-cycle performance evaluation and maintenance decision-making of arch bridges.

## 2. Reliability degradation model considering nonstationary degradation

The time-varying reliability of reinforced concrete arch bridge refers to the reliability of its structural performance changing with time during the service life of the bridge. Because the reinforced concrete arch bridge will be affected by many factors in the long-term use process, such as material aging, environmental erosion, load change, etc. Its structural performance will gradually degrade, leading to the change of reliability. The time-varying reliability theory considers these time-dependent factors, more accurately reflect the safety status and maintenance requirements of reinforced concrete arch bridge structure.

According to the definition of structural reliability, the time-varying reliability of bridge structure considering non-stationary deterioration during service can be expressed as equation (1).


$$P_{rel}(T)=P\left\{R(t)-S(t)>0,\forall t\in (0,T)\right\}$$ (1)

Where: *P*_rel_(*T*) is the time-varying reliability of the bridge structure considering non-stationary deterioration during its service life; *R*(*t*) is the bearing capacity of the target bridge structure at time *t*; *S*(*t*) is the random process of the load effect of the target bridge at time *t*; *t* is the service life of the target bridge structure.

Traditional time-varying reliability analysis models mostly adopt stable degradation schemes. In practical engineering, the degradation process of structural performance is influenced by various random factors and exhibits non stationarity. Therefore, the author refers to reference [16] and introduces a stochastic Gamma process to characterize the non-stationary degradation process of the resistance of reinforced concrete bridge structures. For the load effect variables *S*_*i*_ and *S*_*i*+1_ which obey the extreme value type I distribution, the correlation between load effect variables can be approximately expressed as equation (2).


$$F_{S_{i},S_{i+1}}(x,y)={\left\{\exp\left[-\chi_{i,i+1}\left(\frac{x-\mu_{1}}{\alpha_{1}}\right)\right]+\exp\left[-\chi_{i,i+1}\left(\frac{y-\mu_{2}}{\alpha_{2}}\right)\right]\right\}}^{1/\chi_{i,i+1}}$$ (2)

Where: $F_{S_{i},S_{i+1}}(x,y)$ is the correlation of load variables; *μ*_1_, *μ*_2_, *α*_1_ and *α*_2_ are the parameters of edge distribution *S*_*i*_; $\chi_{i,i+1}$ is the correlation coefficient of adjacent loads.

When the interval between adjacent loads is longer, the correlation between adjacent loads is lower. According to the Bayesian principle, the reliability can be expressed as equation [17] (3).


$$P_{rel}(T)=P(R_{l}>S_{l})\cdot \prod_{i=1}^{n-1}\frac{\int_{\text{0}}^{\infty }\int_{\text{0}}^{\infty }F_{S_{i},S_{i+1}}(r,r-\delta )f_{R_{i}}(r)f_{\Delta_{i+1}}(\delta )drd\delta }{\int_{\text{0}}^{\infty }f_{R_{i}}(r)F_{S_{i}}(r)dr}$$ (3)

Where: *f*_*Ri*_(*r*) is the marginal distribution of resistance *R*_*i*_ in the *i*-th period of service; *r* is the value of the load effect; δ is the load increment for *i* + 1 service periods.

By integrating the aforementioned equations, a time-dependent reliability assessment model for reinforced concrete bridge structures accounting for non-stationary degradation of structural resistance is derived, as presented in Equation (4).


$$P_{rel}(T)=\int_{0}^{\infty }f_{R_{i}}(r)F_{S_{i}}(r)dr\cdot \prod_{i=1}^{n-1}\frac{\int_{0}^{\infty }\int_{0}^{\infty }\left\{1-{\left\{\exp\left[-\chi_{i,i+1}\left(\frac{x-\mu_{1}}{\alpha_{1}}\right)\right]+\exp\left[-\chi_{i,i+1}\left(\frac{y-\mu_{2}}{\alpha_{2}}\right)\right]\right\}}^{1/\chi_{i,i+1}}\right\}f_{R_{i}}(r)f_{\Delta_{i+1}}(\delta )drd\delta }{\int_{0}^{\infty }f_{R_{i}}(r)F_{S_{i}}(r)dr}$$ (4)

According to the definition of time-varying reliability of the structure, the time-varying reliability index of the main arch ring structure is shown in equation (5).


$$\beta (t)=-\phi^{-1}(P_{rel}(t))$$ (5)

Where: *β*(*t*) is the reliability index of the arch ring of reinforced concrete arch bridge in service for t years; $\phi^{-1}$ is the inverse function of the standard normal distribution function.

According to the stress characteristics of the main arch ring, the reinforced concrete arch ring function model is constructed by using the bending bearing capacity, as shown in equation (6).


$$Z(t)=M_{damage}(t)-M(X_{i})$$ (6)

Where: *Z*(*t*) is the main arch ring structure function; *M*_damage_(*t*) and *M*(*X*_*i*_) They are the time-varying bending resistance index considering structural damage and the bending moment effect under random variables.

## 3. Arch ring response surface fitting based on ISSA-Kriging

### 3.1. Principle of improved sparrow optimization algorithm

To enhance the approximation accuracy of the Kriging model and the computational efficiency of structural reliability assessment, this study develops an improved sparrow search algorithm [17–19]. The standard sparrow search algorithm realizes the overall migration of the population to the optimal position in the search space by imitating the social behavior of sparrows foraging and anti predation. The group responsible for foraging in the sparrow population is defined as the explorer, and its position update formula in the *d*-dimensional search space is shown in equation (7).


$$X_{ij}^{t+1}=\left\{ \begin{array}{c} X_{ij}^{t}\cdot \exp(\frac{-i}{r_{\text{1}}\cdot T_{\max}})\;R_{\text{2}}<ST\\ X_{ij}^{t}+Q\cdot L\;R_{\text{2}}\geq ST \end{array}\right.$$ (7)

Where: $X_{worst}^{t}$ is the worst position of the population at the *t*-th iteration; $X_{P}^{t+1}$ is *t*he individual optimal position in the Explorer at the *t* + 1 iteration; *A* is a 1 × *d* matrix composed of 1 or −1 elements, and *A*^+^=*A*^T^(*AA*^T^)^-1^.

Define that there is a safe area in the search space, and a certain number of individuals in the sparrow population are threatened by natural enemies, the position is disturbed according to the fitness value, and the position update disturbance of threatened sparrows is shown in equation (9).


$$X_{ij}^{t+1}=\left\{ \begin{array}{c} Q\cdot \exp\left(\frac{X_{worst}^{t}-X_{ij}^{t}}{i^{\text{2}}}\right)\;i>n/2\\ X_{P}^{t+1}+\left|X_{ij}^{t}-X_{P}^{t+1}\right|\cdot A^{+}\cdot L\;i\leq n/2 \end{array}\right.$$ (8)

Where: $X_{worst}^{t}$ is the worst position of the population at the *t*-th iteration; $X_{P}^{t+1}$ is *t*he individual optimal position in the Explorer at the *t* + 1 iteration; *A* is a 1 × *d* matrix composed of 1 or −1 elements, and *A*^+^=*A*^T^(*AA*^T^)^-1^.

Define that there is a safe area in the search space, and a certain number of individuals in the sparrow population are threatened by natural enemies, the position is disturbed according to the fitness value, and the position update disturbance of threatened sparrows is shown in equation (9).


$$X_{ij}^{t+1}=\left\{ \begin{array}{cc} X_{best}^{t}+\beta \cdot \left|X_{ij}^{t}-X_{best}^{t}\right| & f_{i}>f_{g}\\ X_{P}^{t+1}+r_{\text{2}}\cdot \frac{\left|X_{ij}^{t}-X_{worst}^{t}\right|}{(f_{i}-f_{w})+\varepsilon } & f_{i}=f_{g} \end{array}\right.$$ (9)

Where: $X_{best}^{t}$ is the optimal position of sparrow population at the *t*-th iteration; *β* is a random number subjec*t* to normal distribution, with the mean value of 0 and the variance of 1; *r*_2_ is a random number on [−1, 1]; *f*_i_ is the fitness value of individual *i* of sparrow; *f*_w_ is the worst fitness value of sparrow population; *f*_g_ is the optimal fitness value of the population; ε is the any constant whose denominator is not zero.

Compared with other similar intelligent optimization algorithms, the standard sparrow search algorithm has certain adaptability to high-dimensional optimization problems. However, in practical engineering, there are still some problems such as slow convergence speed and insufficient robustness of optimization. To enhance the optimization performance of the sparrow search algorithm and boost its accuracy and efficiency in addressing the reliability analysis of reinforced concrete arch bridges, this study proposes an Improved Sparrow Search Algorithm (ISSA) incorporating a hybrid strategy. Firstly, the random initialization population strategy is abandoned in the initialization phase, and a tent chaotic mapping strategy is introduced to initialize the population operation. Secondly, the original explorer’s natural constant exponential location update strategy is improved, the location update strategy based on Golden sine section is introduced to update the location. Finally, the Cauchy function is used to perturb the sparrow population and retain the optimal individual.

Tent chaotic map is also called tent map. Compared with deterministic system analysis, tent chaotic map has a relatively uniform distribution function and good correlation in the system [20,21]. Nonlinear chaotic system has natural advantages, which can make the population more ergodic in the search space and improve the convergence speed of the optimal solution. The mapping formula is shown in equation (10).


$$x_{n+1}=f(x_{n})=\left\{ \begin{array}{cc} x_{n}/\alpha & x\in [0,\alpha )\\ \left(1-x_{n})/(1-\alpha \right) & x\in [\alpha ,1) \end{array}\right.$$ (10)

**Fig 1:**
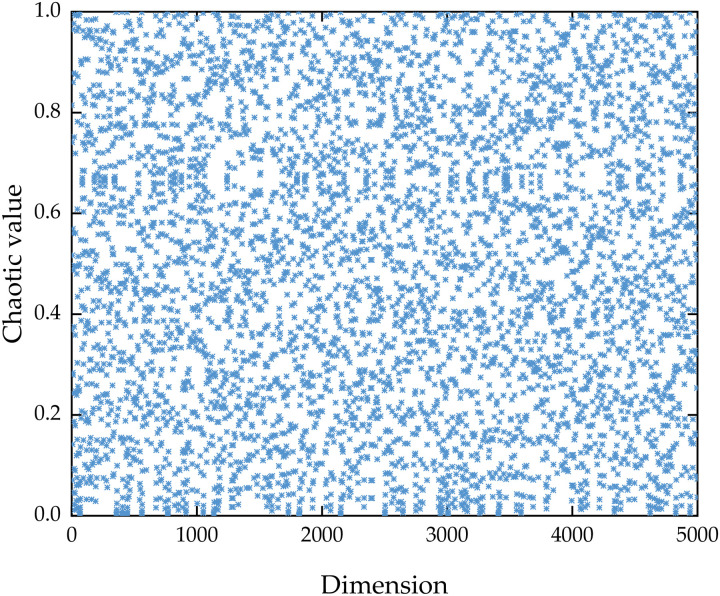
Tent chaotic map distribution.

**Fig 2:**
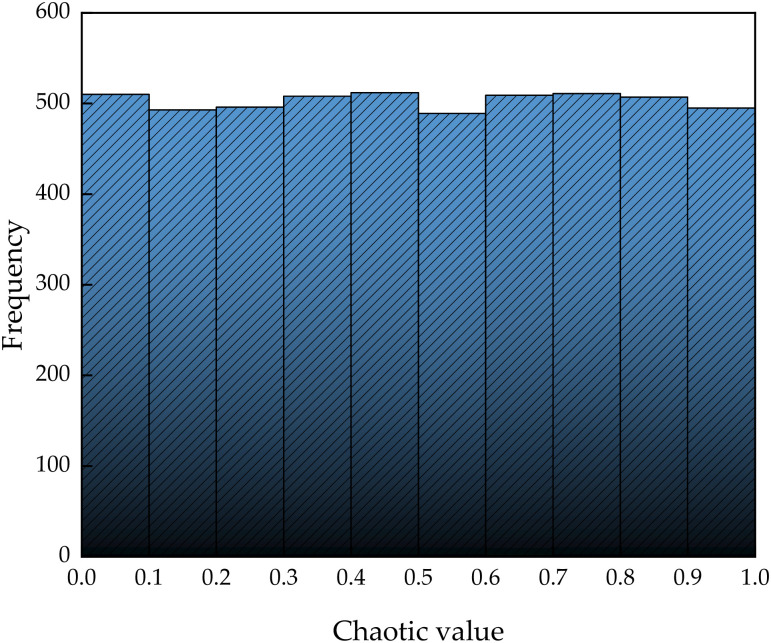
Tent chaotic value distribution interval.

Taking α = 0.499, Fig 1 shows the distribution of tent chaotic map in 5000 dimensions. Fig 1 illustrates that the tent chaotic values lie within the interval [0, 1] and exhibit a uniform distribution. As demonstrated in Fig 2, the frequency of chaotic values across each stage within the [0, 1] interval approximates a uniform distribution pattern.

The scattered points in Fig 1 are evenly distributed throughout the entire area without obvious clustering or blank areas, reflecting the ergodicity of the tent chaotic graph. This indicates that the system can traverse all possible states in its phase space, generate a distribution similar to random, and simulate the unpredictability required for random processes. The bar heights of all sub intervals in Fig 2 are almost uniform, which proves the uniform distribution characteristics of chaotic graphs. The frequencies of each sub interval are similar, ensuring fair sampling and reducing errors caused by uneven distribution in tasks such as random simulation or algorithm initialization.

In order to balance the global search and local development preferences in the parameter optimization process of sparrow population finite element model. Introducing the golden section ratio to improve the explorer’s location update process, as shown in equation (11) and equation (12).


$$X_{ij}^{t+1}=\left\{ \begin{array}{c} X_{ij}^{t}\cdot \left|\sin\gamma_{\text{1}}\right|-\gamma_{\text{2}}\cdot \sin\gamma_{\text{1}}\cdot \left|c_{\text{1}}X_{P}^{t}-c_{\text{2}}X_{ij}^{t}\right|\;\;R_{\text{2}}<ST\\ X_{ij}^{t}+Q\cdot L\;R_{\text{2}}\geq ST \end{array}\right.$$ (11)

$$\left\{ \begin{array}{c} c_{1}=-\pi (1-g)+\pi g\\ c_{2}=-\pi g+\pi (1-g) \end{array}\right.$$ (12)

Where: *γ*_1_ is a random number on [0,2π]; *γ*_2_ is a random number on [0, π]; *C*_1_ and *C*_2_ are the golden section coefficients; *g* is the golden section parameter.

In order to improve the efficiency of sparrow population in the convergence problem of reliability index, the disturbance function of Cauchy distribution is used to perform position mutation operation on the threatened individual, as shown in equation (13).


$$X_{best}^{t*}=\left\{ \begin{array}{c} X_{best}^{t}[1+Cauchy(0,\sigma^{\text{2}})]\;f_{C}>f_{g}\\ X_{best}^{t}\;f_{C}\leq f_{g} \end{array}\right.$$ (13)

Where: *Cauchy*(·) is the standard Cauchy distribution function, and its expression is Cauchy (x)=1/[π (1 + *x*^2^)]; *σ*_2_ is the standard deviation of Cauchy distribution; *f*_C_ is the individual fitness value of new sparrows based on Cauchy disturbance.

The Cauchy function is used to perturb the variation of sparrows, and the greedy retention strategy is adopted for new individuals. The individuals with higher fitness after mutation are retained and the individuals with lower fitness after mutation are discarded to ensure that the sparrow population approaches the optimal region in the search space.

### 3.2. Construction of Kriging agent model

In order to improve the calculation efficiency of time-varying reliability of reinforced concrete arch bridges, Kriging algorithm is used to construct the proxy model of reinforced concrete arch bridges [22–25]. The set of *n* random variables affecting the structural response characteristics of reinforced concrete arch bridges is defined as *X*=(*x*_1_,*x*_2_,..,*x*_*n*_)^T^, the corresponding set of structural feature response variables is *Y*=(*y*_1_,*y*_2_,..,*y*_*n*_)^T^. For the sample set (*x*, *y*) composed of a given arch bridge random variable and response variable, the relationship between the random variable and response variable is established based on Kriging algorithm, as shown in equation (14).


$$y(x)=\sum_{i=1}^{n}\beta_{i}f_{i}(x)+z(x)$$ (14)

Where, *y* (*x*) is the structural response of reinforced concrete arch bridge; *β*_*i*_ is the weight vector of the basis function; *f*_*i*_(*x*) is the basis function; *z*(*x*) is the system deviation.

For the system deviation Z (x) between the actual structural response and the regression model response, it meets the covariance distribution with a mean value of zero, as shown in equation (15).


$$cov[z(x_{i}),z(x_{j})]=\sigma^{2}R(\theta ,x_{i},x_{j})$$ (15)

Where: cov[·] is the covariance function; *R* (·) is a spatial correlation function. In this paper, the surrogate model is constructed by Gaussian function; *θ* is the hyper parameter of spatial correlation function; *σ*^2^ is the variance of random distribution error.

For a given reinforced concrete arch bridge random variable *X* and its response variable *Y*, the mean and variance predicted by Kriging for the sample points to be tested are shown in equation (16) and equation (17) respectively.


$$\mu_{\frac{\mathrm{\backslash buildrel}\mathrm{\backslash lower}3pt\text{\textbackslash{}scriptscriptstyle\textbackslash{}frown\textbackslash{})}}{y}(x)}=f^{T}(xmathord\frac{\mathrm{\backslash buildrel}\mathrm{\backslash lower}3pt\text{\textbackslash{}(\textbackslash{}scriptscriptstyle\textbackslash{}frown\textbackslash{})}}{\beta }+r^{T}(x)R^{-1}(Y-F\frac{\mathrm{\backslash buildrel}\mathrm{\backslash lower}3pt\text{\textbackslash{}(\textbackslash{}scriptscriptstyle\textbackslash{}frown\textbackslash{})}}{\beta })$$ (16)

$$S_{\overset{⏜}{y}\left(x\right)}^{2}=\sigma_{Z}^{2}\left(1-r^{\text{T}}\left(x\right)R^{-1}r\left(x\right)+h^{\text{T}}{\left(F^{\text{T}}R^{-1}F\right)}^{-1}h\right)$$ (17)

Where: *r*(*x*)=[*R*(*x*,*x*_1_),…,*R*(*x*,*x*_n_)]^T^; $\frac{\mathrm{\backslash buildrel}\mathrm{\backslash lower}3pt\text{\textbackslash{}(\textbackslash{}scriptscriptstyle\textbackslash{}frown\textbackslash{})}}{\beta }$ =(*F*^T^*R*^-1^*F*) *F*^T^*R*^-1^*Y*;*h* = *f*(*x*)-*F*^T^*R*^-1^*r*(*x*); $\mu_{\frac{\mathrm{\backslash buildrel}\mathrm{\backslash lower}3pt\text{\textbackslash{}(\textbackslash{}scriptscriptstyle\textbackslash{}frown\textbackslash{})}}{y}(x)}$ is the predicted mean value of the sample points to be tested; $S_{\overset{⏜}{y}\left(x\right)}^{2}$ is the predict variance for sample points to be tested.

In order to achieve the highest fitting accuracy of the surrogate model, it is necessary to solve the spatial correlation function *R* with the smallest expected variance. At this time, the process of establishing the optimal Kriging surrogate model can be transformed into equation (18).


$$\min_{\theta (0}\phi (\theta )={\left|R(\theta )\right|}^{\frac{\text{1}}{m}}\sigma^{\text{2}}$$ (18)

Where: ϕ is the standard normal distribution function; *m* is the number of sample points.

The ISSA algorithm is used to optimize the super parameter *θ* of spatial correlation function, and the structural response surface surrogate model with satisfactory accuracy is established for reliability analysis.

## 4. Time varying reliability analysis process

**Fig 3:**
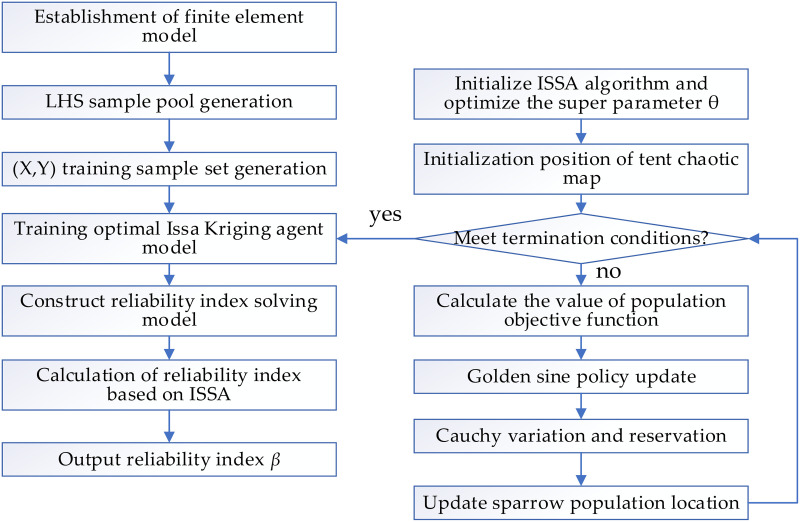
Reliability solution based on ISSA-Kriging model.

Based on ISSA-Kriging structural surrogate model, the reliability analysis of reinforced concrete arch bridge considering material performance degradation is carried out. The basic process is shown in Fig 3.

(1) Determine random variables. Determine the statistical distribution law of structural parameters affecting the structural response characteristics of reinforced concrete arch bridges as the design variables of ISSA-Kriging model [26], as shown in Table 1.

**Table 1:** Statistical characteristics of structural parameters.

Random variable	Mean value	Coefficient of variation	Statistical type
Elastic modulus of arch ring (GPA)	36	0.1	Normal distribution
Elastic modulus of main beam (GPA)	34.5	0.1	Normal distribution
Elastic modulus of column (GPA)	35.5	0.1	Normal distribution
Volume weight of arch ring (kN/m^3^)	27.5	0.1	Normal distribution
Unit weight of main beam (kN/m^3^)	25.5	0.1	Normal distribution
Column bulk density (kN/m^3^)	26.5	0.1	Normal distribution
Phase II dead load (kN/m)	135	0.08	Normal distribution
Vehicle load (kN/m)	55	0.13	Extreme value type I Distribution

(2) Sample point generation. The Latin hypercube sampling method is used to generate the initial sample set *X*={*x*^(1)^, *x*^(2)^,…, *x*^(*N*)^} composed of sample points of structural parameters of reinforced concrete arch bridges [27], regarding the fitting problem of the proxy model for reinforced concrete arch bridges in this article, it was found through experiments that when the sample size is greater than 200, the accuracy of the proxy model tends to stabilize, and the computational efficiency is acceptable at this time. Therefore, the sample size N for this article is 200.
(3) Finite element model establishment. The finite element software is used to establish the numerical calculation model of reinforced concrete arch bridge in different service stages, and the real structural response *Y*={*g*(*x*^(1)^), *g*(*x*^(2)^),…, *g*(*x*^(*N*)^)} corresponding to each sample point in the initial sample set is calculated based on the finite element model [28].
(4) Optimal Kriging model training. Based on the {*X*, *Y*} sample set of the initial experimental design, the standard Kriging model is trained. Taking equation (18) as the objective function, the hyper parameter *θ* of Kriging model is compiled into the position information of ISSA, and the optimal Kriging structure proxy model is trained.
(5) Model accuracy verification and reliability index solution. *R*² and *RMSE* are employed to assess the accuracy of the surrogate model, while solving the reliability index is transformed into minimizing the distance from the origin to the limit state surface in the standard normal coordinate system, as defined in Equation (19). This optimization problem is then addressed using the ISSA algorithm.

$$\left\{ \begin{array}{c} \underset{u^{*}}{\min}\beta =\min\left(\sqrt{\sum_{i=1}^{n}u_{i}^{2}}\right)\\ s.t.\;g(x^{*})=g\left(F^{-1}\left(\Phi (u^{*})\right)\right)=\varepsilon \end{array}\right.$$ (19)

Where: *β* is the reliability index; u* is the design check point of standard normal distribution space; *g*(*x*) is the limit state function; *F*^-1^(*x*) is the inverse function of the probability density function of random variables; *ε* is convergence error, this paper takes 0.001.

## 5. Time varying reliability analysis process

### 5.1 Engineering background and finite element model

**Fig 4:**
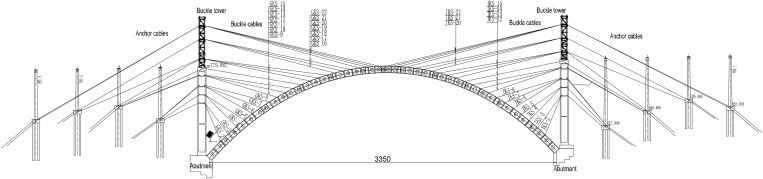
Layout of bridge type(unit: dm).

Shuiluohe Bridge is a deck reinforced concrete cantilever cast arch bridge, the main arch adopts constant section catenary hingeless arch, with a calculated span of 335m, a rise span ratio of 1/4.2, and an arch axis coefficient of 1.8. The arch ring is constructed in 45 segments. The arch foot is cast-in-place with scaffolding, the closure segment is constructed with hangers, and other segments are cast with hanging basket cantilever, the layout of bridge type is shown in Fig 4.

**Fig 5:**
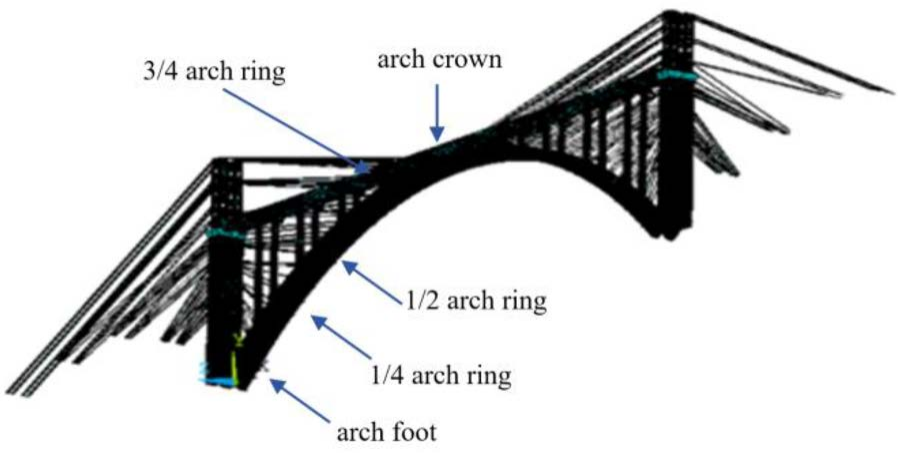
Finite element model.

The finite element software is used to establish the bridge structure calculation model, in which the truss element is used for the buckle cable and anchor cable, the beam element is used for the buckle tower, and the solid element is used for the concrete arch ring. The finite element model is shown in Fig 5.

### 5.2. Algorithm performance verification

Four benchmark functions with varying dimensions are employed to evaluate the convergence performance of the Improved Sparrow Search Algorithm (ISSA) incorporating a hybrid improvement strategy. Additionally, the standard Sparrow Search Algorithm (SSA) and Particle Swarm Optimization (PSO) are introduced for horizontal performance comparison. All algorithms are configured with a population size of 40, a maximum iteration count of 500, and a function dimension set to 20. The baseline algorithm parameters are detailed in Table 2 and 3.

**Table 2:** Test Function Information.

Function number	Function name	Optimal solution
F1	Sphere	0
F2	Rastrigin	0
F3	Ackley	0
F4	Griewank	0

**Table 3:** Algorithm parameter settings.

Algorithm name	Parameter setting
PSO	*c*_1_ = *c*_2_ = 2; *ω*_max_ = 0.9; *ω*_min_ = 0.4
SSA	The proportion of explorers is 20%; The proportion of threatened individuals is 20%; ST = 0.8
ISSA	*α* = 0.499; *g* = 0.618; *Cauchy*~*C*(1,0). Other parameters are the same as above

Each algorithm runs independently for 30 times, and the average value (AVG), optimal value (best) and standard deviation (STD) are used to measure the optimization accuracy and stability of each algorithm, Table 4 shows the optimization results of each algorithm under different test functions.

**Table 4:** Algorithm performance test results.

Function number	Algorithm	AVG	BEST	STD
F1	PSO	0.00	0.00	0.00
	SSA	0.00	0.00	0.00
	ISSA	0.00	0.00	0.00
F2	PSO	5.49E-39	6.77E-42	1.94E-34
	SSA	8.44E-44	7.17E-62	3.82E-43
	ISSA	4.81E-65	3.58E-73	1.97E-81
F3	PSO	6.71E-31	2.57E-41	5.98E-35
	SSA	5.63E-38	7.29E-46	3.73E-39
	ISSA	4.87E-49	8.57E-71	6.05E-61
F4	PSO	6.87E-11	3.78E-17	4.54E-09
	SSA	4.92E-10	2.55E-16	5.71E-12
	ISSA	7.18E-26	6.75E-38	4.73E-32

From the optimization results of F1 function, it can be seen that the average value, optimal value and standard deviation of all algorithms are 0, indicating that the three algorithms can better complete the optimization of F1 function.

From the optimization results of F2, F3 and F4 functions, the optimization ability of ISSA for complex functions is much higher than that of PSO and SSA algorithms, and the optimal value and average value are closer to the global optimal solution, in addition, the standard deviation of ISSA algorithm is smaller, which shows that Issa algorithm has higher stability for solving functions.

By comparing different performance indexes of different test functions, it is found that the improved initial population strategy and population updating method greatly improve the overall convergence performance of ISSA algorithm, making it more suitable for the parameter optimization of Kriging model and the reliability solution of arch bridge structure.

### 5.3. Feasibility of ISSA algorithm in reliability calculation

To verify the superiority of the ISSA algorithm in reliability analysis, a comparative analysis was conducted on the reliability of the arch foot and arch top at the beginning of service. The standard SSA algorithm and Monte Carlo method were used for comparison, and the calculation results are shown in Table 5. According to Table 5, the reliability calculated by Monte Carlo method is the theoretical value. Both SSA and ISSA algorithms can complete the reliability calculation of arch foot and arch crown. However, ISSA algorithm benefits from the advantage of improved strategy, with smaller relative error and higher calculation accuracy. The relative error of arch foot reliability is only −0.15%, and the relative error of arch crown reliability is only −0.13%, which is much higher than SSA algorithm.

**Table 5:** Comparison of Reliability Solution Methods.

solving algorithm	Arch foot reliability	relative error	arch crown reliability	relative error
Monte Carlo method	4.559	/	4.563	/
SSA	4.451	−2.37%	4.466	−2.13%
ISSA	4.552	−0.15%	4.557	−0.13%

### 5.4. Agent model training and validation

The finite element software is employed to compute the theoretical structural response of reinforced concrete arch bridges, and a sample set is generated via Latin hypercube sampling. To validate the approximation accuracy of the proposed ISSA-Kriging surrogate model for the structural response of such bridges, a BP neural network and the Kriging model are introduced as comparative models to train the sample set.

**Fig 6:**
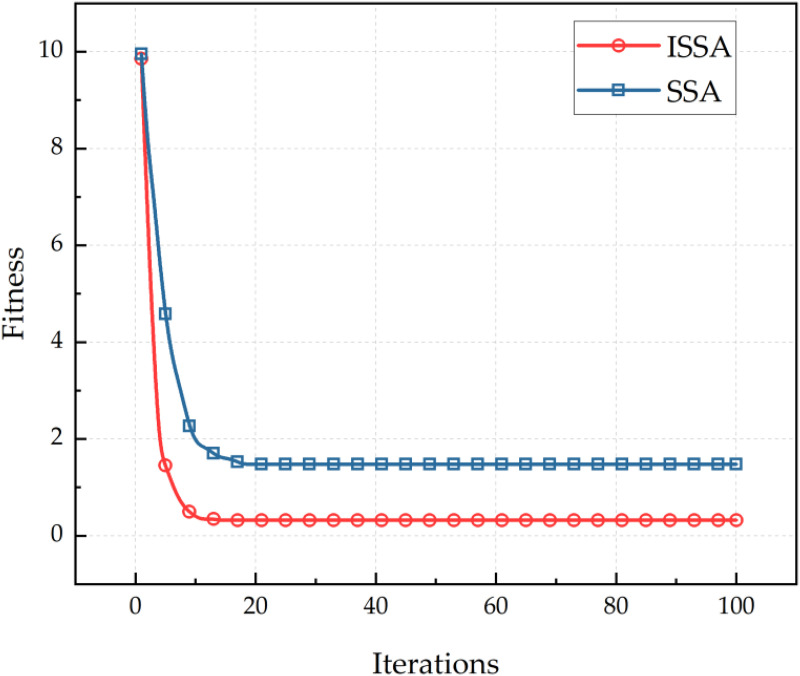
Convergence curve of sparrow search algorithm.

Fig 6 shows the convergence curve of ISSA algorithm for Kriging model parameter optimization. As can be seen from Fig 6, ISSA algorithm has better adaptability to the Kriging model parameter optimization problem than SSA algorithm, and its population convergence speed is faster, and the convergence accuracy is higher, which is more conducive to the training performance of Kriging model.

**Fig 7:**
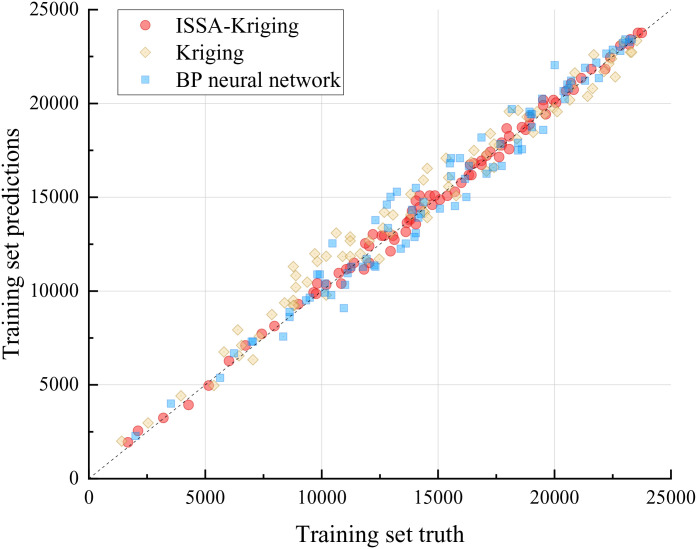
Training set fitting results.

**Fig 8:**
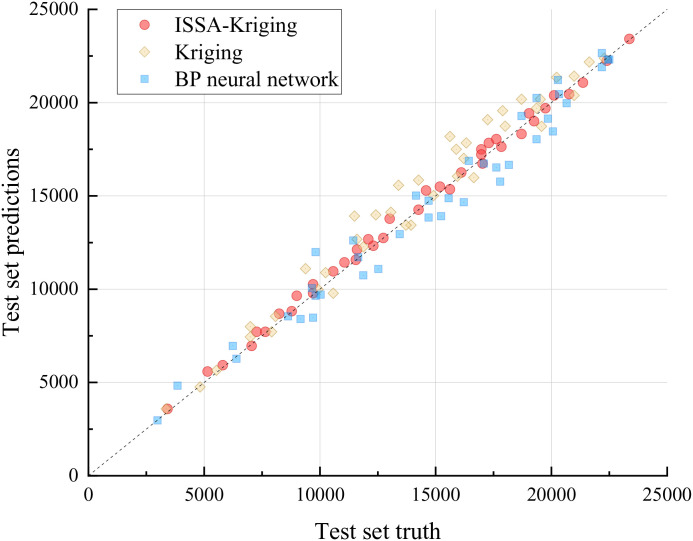
Test set fitting results.

Figs 7 and 8 demonstrate that the ISSA-Kriging model exhibits superior predictive capability for the structural responses of reinforced concrete arch bridges subject to diverse random variables, with predicted points closely aligning with the true response trajectory. In contrast, both the Kriging model and BP neural network exhibit notable discrepancies in predicting structural responses, showing poorer alignment with the true response trajectory.

**Fig 9:**
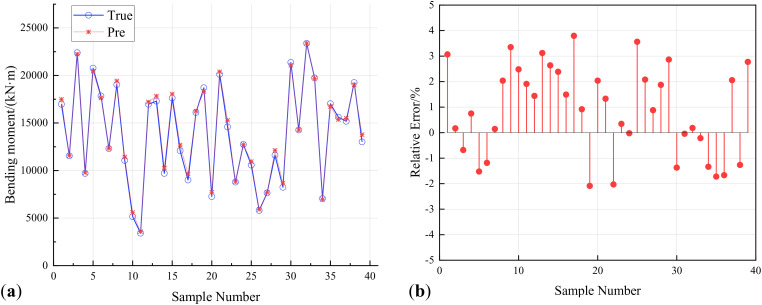
ISSA-Kriging model validation. (a) Predicted response results of ISSA-Kriging model. (b) Prediction relative error of ISSA Kriging-model.

**Fig 10:**
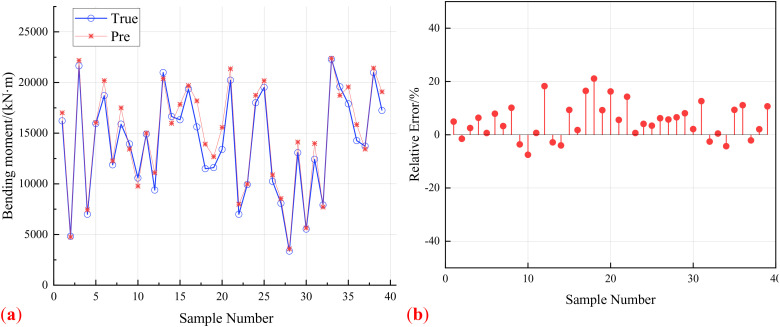
Kriging model validation. (a) Predicted response results of Kriging model. (b) Prediction relative error of Kriging model.

**Fig 11:**
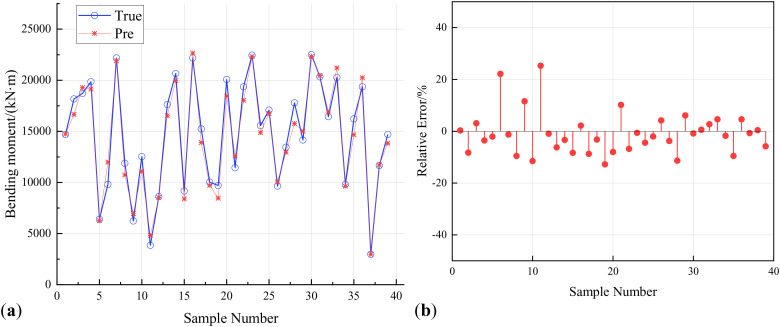
BP neural network model validation. (a) Predicted response results of BP neural network model. (b) Prediction relative error of BP neural network model.

Extract 40 sample points to verify the prediction error of various machine learning models on the response surface of reinforced concrete arch bridges. Figs 9, 10, 11 show the prediction results and relative errors of ISSA-Kriging, Kriging and BP neural network models respectively.

Fig 9 illustrates that the ISSA-Kriging model demonstrates excellent fitting performance for the validation samples. The relative error of the 40 validation set samples extracted by repeated random sampling is within 4%, and the average absolute relative error is only 1.66%. It shows that ISSA-Kriging model not only has strong fitting ability for training set samples, but also retains high generalization ability, and has high prediction accuracy for random sampling validation samples.

Fig 10 shows that the Kriging model exhibits significant discrepancies in predicting validation samples, with the relative prediction error of some samples exceeding 20%. Moreover, the average absolute relative error across all test points is 6.66%, indicating that its structural response predictions suffer from substantial inaccuracies.

Fig 11 demonstrates that the BP neural network model exhibits marginally higher prediction accuracy than the standard Kriging model; however, it shows substantial error standard deviation and significantly inferior generalization ability compared to the ISSA-Kriging model, with an average absolute relative error of 5.98% across all validation points.

Table 6 presents the model accuracy index results for each machine learning surrogate model. As indicated in Table 6, the ISSA-Kriging model significantly outperforms the standard Kriging model and BP neural network model across all accuracy metrics. Its RMSE and MAE indices are substantially lower than those of other models, demonstrating that optimizing the hyperparameters of machine learning models using an optimization algorithm can effectively enhance their generalization performance. While standard models may adapt well to training samples, they exhibit weak generalization ability, leading to large prediction errors for unknown data points.

**Table 6:** Model accuracy verification results.

Model name	*R*^2^(95%CI)	RMSE(95%CI)	MAE(95%CI)
ISSA-Kriging	0.99(0.98-1.00)	360.84(345.21-376.47)	294.95(280.12-309.78)
Kriging	0.96(0.94-0.98)	1116.69(1080.33-1153.05)	884.41(850.22-918.60)
BP neural network	0.97(0.95-0.99)	951.44(920.55-982.33)	768.78(740.15-797.41)

### 5.5. Time varying reliability analysis of arch ring

**Fig 12:**
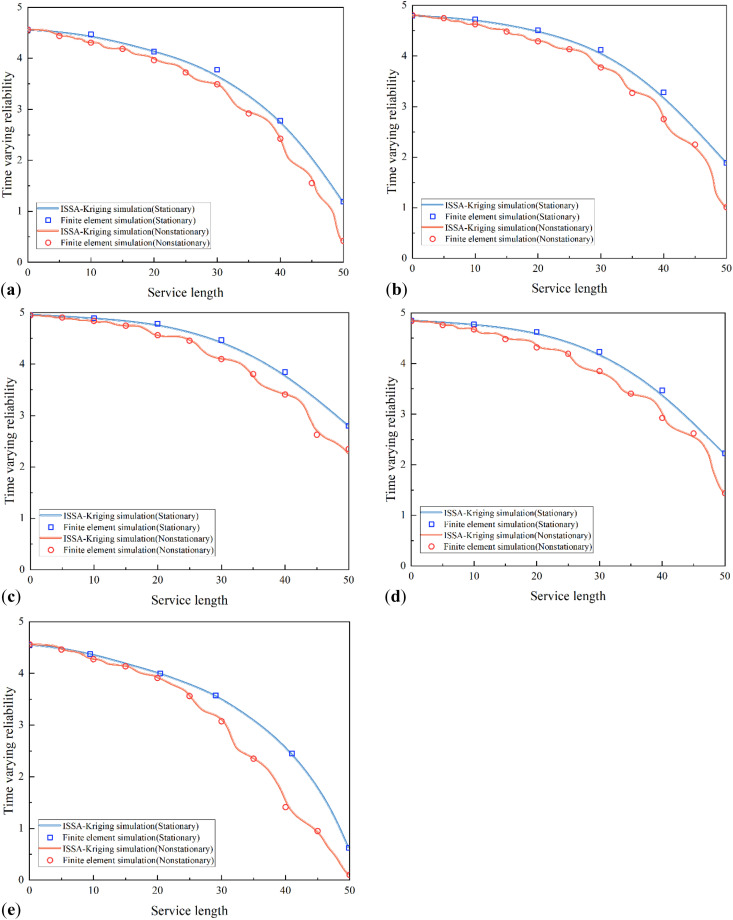
Time varying reliability of arch ring. (a) arch foot. (b) 1/4 arch ring. (c) 1/2 arch ring. (d) 3/4 arch ring. (e) arch crown.

Calculate the time-varying reliability curves of reinforced concrete arch bridge structural components under conventional and non steady state degradation based on ISSA Kriging surrogate model and finite element model, respectively. Considering structural symmetry, a semi-structured model is chosen for analysis. Fig 12 shows the time-dependent reliability degradation results of the arch foot, 1/4 arch ring, 1/2 arch ring, 3/4 arch ring, and arch crown section of the main arch half structure.

Fig 12 shows that as the usage time increases, the time-varying reliability of the reinforced concrete arch bridge arch ring gradually deteriorates. The structural reliability of the main arch ring monotonically decreases, and the rate of decrease accelerates over time, indicating that the later stages of its service life correspond to more severe degradation of the bridge structure. Compared with the time-varying reliability curve under traditional nonlinear steady degradation, the steady state curve shows a gradually decreasing trend, while the non-stationary state curve not only exhibits the characteristic of fluctuating decrease, but also the time-varying reliability index decreases faster. This phenomenon indicates that non-stationary degradation factors accelerate the reliability degradation of arch ring structures, causing them to face safety risks earlier in their service life. It also highlights the limitations of traditional assumption of steady degradation in practical engineering safety assessment.

The analysis of the time-varying reliability of arches at different stages of use shows that during early use, the prediction based on the stationary degradation time-varying reliability model is very consistent with the prediction based on the non-stationary degradation model. This indicates that when the structure maintains a relatively good operating state, the impact of non-stationary load effects on structural safety is minimized. However, over time, the non-stationary degradation effect significantly reduces the structural reliability of the arch ring, and its impact monotonically increases with increasing usage time.

From the perspective of model validity verification, the simulation results of the ISSA Kriging model in both stationary and non-stationary degradation scenarios maintain good consistency with the finite element simulation results. Both the overall attenuation trend of the curve and the range of numerical changes in reliability are highly consistent. This result validates the accuracy of the ISSA Kriging model in predicting the time-varying reliability of reinforced concrete arches, indicating that the model can effectively capture the structural reliability evolution laws under stationary and non-stationary degradation mechanisms, providing a reliable numerical tool for subsequent large-scale parameter analysis and engineering optimization.

**Fig 13:**
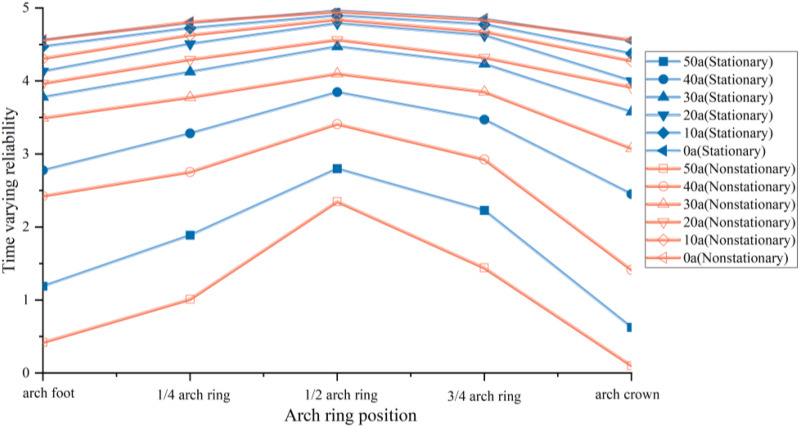
Time varying reliability of different arch ring segments.

Fig 13 illustrates the variation patterns of reliability indices for each arch segment under different service durations, revealing significant spatiotemporal differences in the reliability of the main arch. To rigorously verify these differences, we conducted an independent-samples t-test on the reliability indices under stationary and non-stationary degradation at the key service time point (50th year). Results show that the p-value is less than 0.05, confirming a statistically significant difference between the two degradation scenarios.

Under both stationary and non-stationary degradation scenarios, the reliability of the semi-structure of the main arch ring exhibits an obvious “unimodal” distribution characteristic, with higher reliability indices in the middle region and lower ones in the side regions. Specifically, the reliability indices of the arch springing and arch crown are significantly lower, while those in the area near the mid-span (1/2 arch ring) are relatively higher. This spatial distribution is consistent with the specifications mentioned in the <Code for Durability Design of Concrete Structures> (GB/T 50476−2008) [29], which points out that spatial differences in stress concentration will lead to non-uniform degradation of arch structures. In engineering practice, the middle region of the arch rib usually has a more favorable stress state, whereas the arch springing and arch crown often experience higher stress due to their mechanical characteristics, resulting in more severe degradation and lower reliability indices.

When the service duration extends from 0 to 50 years, the reliability index of the mid-span arch segment under stationary degradation decreases from 4.96 to 2.79, with a reduction rate of approximately 43.7%. In contrast, under non-stationary degradation, this index drops to 2.35, with a reduction rate of approximately 52.6%. By comparing the degradation trends of the arch ring’s reliability indices, it can be seen that the degradation rate of the arch ring’s reliability under non-stationary degradation exceeds the range of empirical degradation rates in long-term reliability assessments of main arch rings of reinforced concrete arch bridges [30]. This difference highlights that non-stationary loads exacerbate the accumulation of structural damage, thereby accelerating the aging process of reinforced concrete structures.

**Fig 14:**
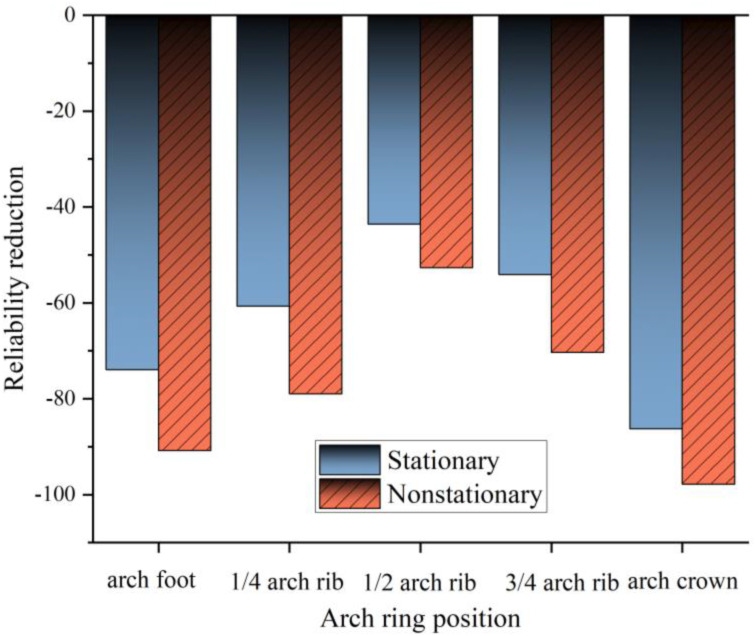
Reliability reduction of arch ring after 50 years of service.

Fig 14 illustrates the reduction in reliability indices of different arch ring segments after 50 years of service. Analysis of the figure reveals that the reliability reduction under non-stationary degradation generally exceeds that under stationary degradation, with particularly pronounced declines observed at the arch foot and arch crown segments. These findings highlight the necessity of accounting for non-stationary degradation characteristics in the design and maintenance of reinforced concrete arch bridge arch rings. Greater emphasis should therefore be placed on monitoring the reliability of arch foot and arch crown segments, with targeted maintenance and reinforcement measures implemented as needed.

## 6. Conclusions

To evaluate the structural reliability of reinforced concrete arch bridges under non-stationary degradation, this study first derived a reliability calculation method for non-stationary degradation scenarios, and then proposed a reliability calculation model based on the Improved Sparrow Search Algorithm-Kriging (ISSA-Kriging) that is suitable for such bridges. The model was validated and analyzed using the Shuiluo River Bridge as an engineering case, and the key research findings and relevant discussions are as follows:

(1) The ISSA-Kriging surrogate model exhibits high prediction accuracy: In repeated random sampling tests, its sample response error is controlled within 4%, and its accuracy is approximately 60% higher than that of the standard Kriging model. Comparison with finite element analysis results shows that this model can reliably fit the time-varying reliability curves of the main arch ring of reinforced concrete arch bridges, confirming its applicability in large-scale parametric analysis and engineering optimization.
(2) Under the stationary degradation scenario, the time-varying reliability of the main arch ring shows a gentle downward trend; in contrast, under the non-stationary degradation scenario, the reliability curve exhibits a “fluctuating decline” characteristic, and the decay rate of the time-varying reliability index accelerates significantly. As the service life extends, the impact of non-stationary degradation on structural reliability shows a monotonically increasing trend, highlighting its key role in long-term safety assessment.
(3) Compared with stationary degradation, non-stationary degradation accelerates the decay rate of the reliability index of the main arch ring by 20%–30%. After 50 years of service, under non-stationary degradation, the reliability reduction rates of the arch springing, arch crown, and mid-span (1/2 arch ring) reach 90.8%, 97.8%, and 52.7% respectively, resulting in an obvious “unimodal” reliability distribution in the semi-structure of the main arch ring. This spatial difference, combined with the exacerbating effect of non-stationary load fluctuations on the accumulation of structural damage, indicates that targeted durability protection for key components is necessary during the design and maintenance phases.

This study has limitations that require attention: Although the constructed non-stationary degradation model conforms to the law of non-stationary degradation in theory and its rationality has been verified through numerical simulation, it lacks direct calibration with long-term on-site monitoring data. This leads to a certain deviation between the model parameters and the actual degradation state of structures in practical engineering, and its practical applicability needs further verification.

Future research will focus on “integrating structural health monitoring data”: By acquiring structural health monitoring data during the long-term operation of target arch bridges, key characteristic parameters of non-stationary degradation will be extracted to dynamically revise the existing non-stationary degradation model. At the same time, monitoring data will be used to update the load fluctuation coefficients and degradation rate parameters in the model in real time, enabling the model to more accurately reflect the actual degradation process of structures. This will bridge the gap between numerical simulation and actual structural performance, thereby improving the engineering practical value of non-stationary reliability assessment results.
